# Effect of Maltodextrins on the Rheological Properties of Potato Starch Pastes and Gels

**DOI:** 10.1155/2013/869362

**Published:** 2013-05-20

**Authors:** Lesław Juszczak, Dorota Gałkowska, Teresa Witczak, Teresa Fortuna

**Affiliations:** ^1^Department of Analysis and Evaluation of Food Quality, University of Agriculture in Krakow, Balicka 122, 30-149 Krakow, Poland; ^2^Department of Engineering and Machinery for Food Industry, University of Agriculture in Krakow, Balicka 122, 30-149 Krakow, Poland

## Abstract

The study examines the effects of maltodextrins saccharified to various degrees on some rheological properties of potato starch dispersions. Pasting characteristics, flow curves, and mechanical spectra were determined for native potato starch and for its blends with potato maltodextrins having dextrose equivalents (DE) of 10.5, 18.4, and 26.5. The results showed that medium-saccharified maltodextrin (DE = 18.4) gave the strongest effect, manifesting itself as a considerable reduction in the viscosity at pasting, a decrease in apparent viscosity during flow, and a decrease in the storage and loss moduli. Addition of high-(DE = 26.5) or low-(DE = 10.5) saccharified maltodextrins had a markedly smaller effect on the rheological properties of starch. The differences in the effects produced by the maltodextrins are closely connected to the degree of polymerisation of the maltooligosaccharides in the systems.

## 1. Introduction

Starch, one of the most common polysaccharides, has a number of specific properties that make it highly useful in the food industry and other sectors of the economy. It belongs to the cheapest thickening agents, texturizers, filling agents, and stabilizers. Heating starch granules in water environment causes them to paste. When they release amylose in the process, starch loses its specific granular structure [1–3]. The characteristic pasting temperature depends on botanical origin of starch as well as on the presence of other substances in the system. Sugars present in a starch suspension reduce the water activity of the system and stabilize the amorphous regions of the granules. As a result, starch pasting temperature increases and rheological properties of the system change [4–7]. The greater this effect, the higher the concentration of the solution and the greater the molecular weight of the substance added [3, 8, 9].

In the temperate climatic zone countries, such as Poland, potatoes are an important source of starch. Potato starch differs from cereal starches mainly in the size and structure of granules [10, 11], amylose content, phosphorus content, and manner of phosphorus bonding, as well as in fat and protein contents [1, 12–14]. Since natural properties of starch are not always advantageous in terms of technology and application, it is often subjected to various modifications, among them hydrolysis. Such a modification may produce maltodextrins, that is, carbohydrate polymers built of D-glucose units having a dextrose equivalent (DE) of under 20 [15, 16]. In Poland, starch hydrolysates with a DE of 20 to 30 are called high-saccharified maltodextrins. Due to their properties, maltodextrins are widely applied in the food industry [15]. One of the interesting issues is the influence of maltodextrins on starch polymers. For example, Smits et al. [17] observed that the presence of maltooligosaccharides with polymerization degrees (DP) of 2 to 5 hinders the formation of amylose helices, thus reducing the retrogradation degree of wheat starch, while those with a DP exceeding 6 may form by themselves small helices that co-crystallise with starch polymers, thus accelerating retrogradation. An increase in the level of retrogradation of starch at temperature of 2°C in the presence of high-molecular-weight maltooligosaccharides has been reported by Wang and Jane [18]. As found by Durán et al. [19] adding oligosaccharides with DP of 3 to 5 delays the gelatinisation of starch and reduces the enthalpy of its retrogradation. Such a phenomenon may be used for inhibiting the staling of bread [17, 20–22].

Knowledge of the rheological properties of starch pastes and gels is of vital importance to the food industry and other sectors utilizing starches as a raw material [3, 22]. Since in complex food systems starch coexists with a wide range of other compounds, it is useful to understand the influence of individual components of foods on the properties of starch. The present study was designed to determine the effect of maltodextrins of different dextrose equivalents (called low-, medium-, and high-saccharified maltodextrins) on chosen rheological properties of potato starch.

## 2. Materials and Methods

Potato starch was obtained from PZZ Piła, Poland, and potato maltodextrins were provided by CLPZ Luboń, Poland. The maltodextrins were saccharified to different degrees: low (DE = 10.5, DP = 10.6), medium (DE = 18.4, DP = 6.0), and high (DE = 26.5, DP = 4.2). The dextrose equivalent (DE) was determined by Lane-Eynon's method according to the relevant Polish Standard (PN-EN ISO 5377:2001). The mean degree of polymerization (DP) was calculated on the basis of dextrose equivalent values: DP = 111/DE.

Rheological studies were conducted at constant concentration of starch (5 g d.w./100 g). The starch-maltodextrin systems were produced by dissolving an appropriate amount of maltodextrins (1, 2, or 3 g d.w./100 g) in distilled water and then adding starch.

The pasting characteristics of both native starch and starch-maltodextrin blends were determined in a Brabender viscograph, type 801201 (Germany) with a measuring cup of 250 cmg at a rotation speed of 75 rpm. The systems studied were heated and then cooled at a rate of 1.5°C/min using the following procedure: raising temperature from 25 to 96°C, maintaining constant temperature of 96°C during 20 minutes, reducing temperature from 96 to 50°C, and maintaining constant temperature of 50°C during 10 minutes. The viscograms obtained were used to read pasting temperature, peak viscosity, temperature at peak viscosity, viscosity at 96°C, viscosity after 20 minutes at 96°C, viscosity at 50°C, and viscosity after 10 minutes at 50°C.

Samples for rheometric investigations were prepared by heating the suspension of starch or starch with each maltodextrin at temperature of 95°C for 30 minutes while stirring it continuously at a rate of 250 rpm. Next the hot paste was placed in the measuring element of the rheometer, relaxed, and thermostated during 15 minutes at the temperature of measurement. Flow curves at 50°C were obtained by using a rotational rheometer Rheolab MC1 (Physica, Germany) with a coaxial cylinders system (cup diameter 27.12 mm, bob diameter 25.00 mm) for the shear rate range of 1–300 s^−1^. The experimental curves were described employing Herschel-Bulkley equation:

(1) $$\begin{array}{c} \tau =\tau_{0}+K\cdot {\dot{\gamma }}^{n}, \end{array}$$

where *τ* is the shear stress (Pa), $\dot{\gamma }$ is the shear rate (s^−1^), *τ*_0_ is the yield stress (Pa), *K* is the consistency coefficient (Pa · s^*n*^), and *n* is the flow behavior index.

Mechanical spectra at 25°C were determined by using a Rheostress RS rheometer (Haake, Germany) with a cone-plate system (cone diameter 35 mm, angle 2°, gap width 0.105 mm). The measurements were made in the linear viscoelasticity range at a constant strain of 0.03 in the frequency range of 0.1–10 Hz.

Statistical assessment was done by performing a one-way analysis of variance and calculating the least significant difference (LSD) at *α* = 0.05.

## 3. Results and Discussion

**Figure 1:**
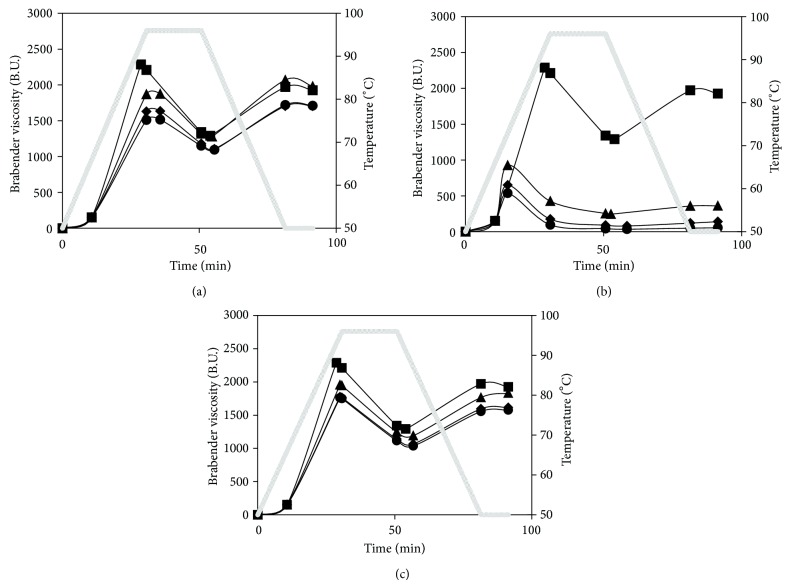
Pasting curves of native starch and blends with (a) low-saccharified maltodextrin, (b) medium-saccharified maltodextrin, (c) high-saccharified maltodextrin. Maltodextrin concentration: 0—□, 1—∆, 2—*◊*, 3—○ g/100g.

Figure 1 shows the pasting curves of native potato starch and starch-maltodextrin systems, and Table 1 provides the pasting characteristics. Maltodextrins added to starch altered its viscosity at pasting. The changes depended on the kind of maltodextrin and its amount in the system. They did not have any influence on the pasting temperature of starch, except for low-saccharified maltodextrin added in the amount of 3 g/100 g, in which case this temperature slightly (by 1.5°C) but significantly increased (Table 1). Low-saccharified maltodextrin blended with starch brought about a marked fall in peak viscosity which was increasing with maltodextrin content in the system. In addition, such systems reached peak viscosity at a slightly higher temperature than native starch (Table 1). Potato starch is characterized by significantly higher values of peak viscosity as compared to cereal starches, that is, due to its high swelling capacity at relatively low temperature [14]. The presence of maltodextrins in the system reduces swelling capacity of the starch due to restriction of the amount of water available for starch granules, in the way depending on a DE of maltodextrin. Maltodextrins with low DE and thus with high DP values can also swell, however, to a lower degree than the native starch granules. High-saccharified maltodextrins swell to a low degree but more easily solubilize and thus thicken the continuous phase of the system. In the present study, the viscosity at 96°C of the systems containing low-saccharified maltodextrin was significantly decreased compared to starch paste. Maintaining the pastes at that temperature caused a sharp fall in viscosity both for native starch and the blends. The system with low-saccharified maltodextrin added at a level of 1 g/100 g displayed similar viscosity to that of the paste of native starch, while at higher maltodextrin levels the viscosity of the systems was significantly reduced. Similarly, at cooling, the viscosity of the system containing the smallest amount of low-saccharified maltodextrin did not differ from that of the native starch paste, while adding a greater amount of maltodextrin caused the viscosity of the pastes to decrease. No differences in viscosity were observed between the systems with 2 and 3 g/100 g maltodextrin. The fall in paste viscosity due to the addition of maltodextrin was the most pronounced for medium-saccharified one and was larger when maltodextrin content was higher (Figure 1(b), Table 1). What is more, the systems containing this kind of maltodextrin reached peak viscosity at much lower temperature (72.3–73.0) than the other systems. High-saccharified maltodextrin (Figure 1(c), Table 1) also significantly reduced the peak viscosity of the paste, but to a much smaller degree than medium-saccharified one. The systems containing 2 and 3 g/100 g of the maltodextrin in question showed similar viscosity. The peak viscosity and the viscosity at 96°C were higher for the systems with high-saccharified maltodextrin than for the corresponding systems with low-saccharified maltodextrin, while after cooling, that pattern became reversed. The final viscosity of the starch paste results from a structure of two-phase gel-like system formed after cooling stage, in which the continuous phase is composed of associated linear amylose chains, while the dispersed phase is made of fragments of starch granules consisted mainly of amylopectin. The process of the association of the linear amylose chains is an initial stage of the retrogradation phenomenon. According to the literature data [19, 20], low-saccharified maltodextrins, that is, with higher DP, can be involved in forming the structures of the continuous phase, while medium-saccharified maltodextrins with medium-length chains are too small for cocreation of the gel-like structures; however, they have enough long chains in order to restrict amylose association and weaken the structure of the system.

**Figure 2:**
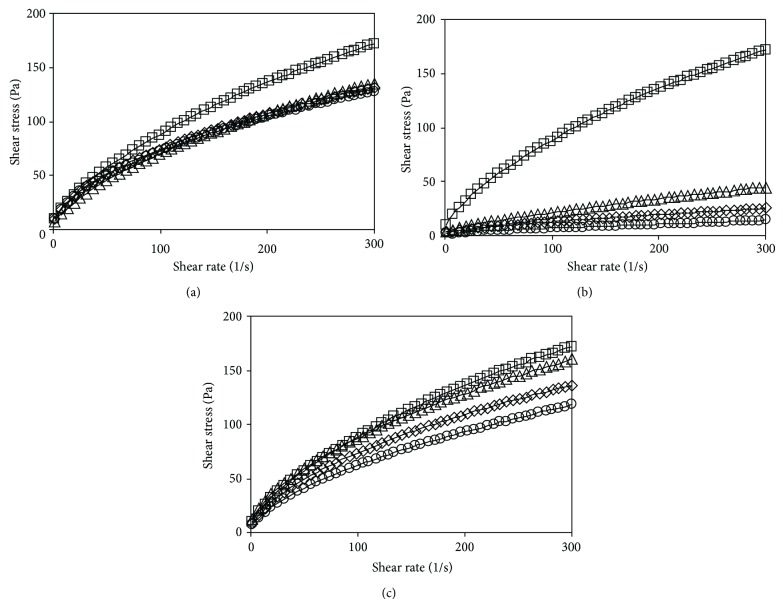
Flow curves of native starch and blends with (a) low-saccharified maltodextrin, (b) medium-saccharified maltodextrin, (c) high-saccharified maltodextrin. Maltodextrin concentration: 0—□, 1—∆, 2—*◊*, 3—○ g/100g.

Figure 2 shows the flow curves of native starch and its blends with maltodextrins. The experimental curves were described using the parameters of Herschel-Bulkley model (Table 2). Addition of low-saccharified maltodextrin resulted in reduced shear stresses, especially at higher shear rates (>50 s^−1^) (Figure 2(a)). The flow curves of starch-low-saccharified maltodextrin systems were similar for all amounts of the maltodextrin added. The pastes with low-saccharified maltodextrin exhibited lower values of the yield stress than the paste of native starch and, except for a system with 1 g of maltodextrin per 100 g, smaller values of the flow behavior index (Table 2). The consistency coefficient decreased for the latter system and increased for the others. According to the pasting curves, the effect of maltodextrins on the rheological properties of starch pastes was the largest for the pastes containing medium-saccharified maltodextrin. In this case the flow curves showed a considerable reduction in shear stresses, as compared to the paste of native starch (Figure 2(b)). The greater the decrease, the higher was the amount of maltodextrin in the system. The same was true for the yield stress and the consistency coefficient of these systems (Table 2). In contrary, values of the flow behavior indices of these systems were markedly greater than those of the native starch paste; however, they were not significantly dependent on the amount of maltodextrin. The presence of the high-saccharified maltodextrin at a level of 1 g/100 g caused a rise in the values of the yield stress and the consistency coefficient and a significant decrease in the values of the flow behavior index (Table 2). When the level of the high-saccharified maltodextrin was increased, the yield stress of the paste decreased, as did the consistency coefficient. There were no significant differences in the flow behavior indices between the systems containing different amounts of the maltodextrin (Table 2). During shearing of the starch paste, the destruction and the following reconstruction of its structure take place. The presence of maltodextrins in the starch pastes affected in a different way their flow behavior. Similarly to the pasting characteristic, the medium-saccharified maltodextrins with medium DP value had the greatest effect on the flow behavior of the starch pastes. It results presumably from the length of the maltodextrin chains, which are too short in order to cocreate the structure of starch paste but enough long in order to disturb formation of the continuous phase consisting of the linear amylose. 

**Figure 3:**
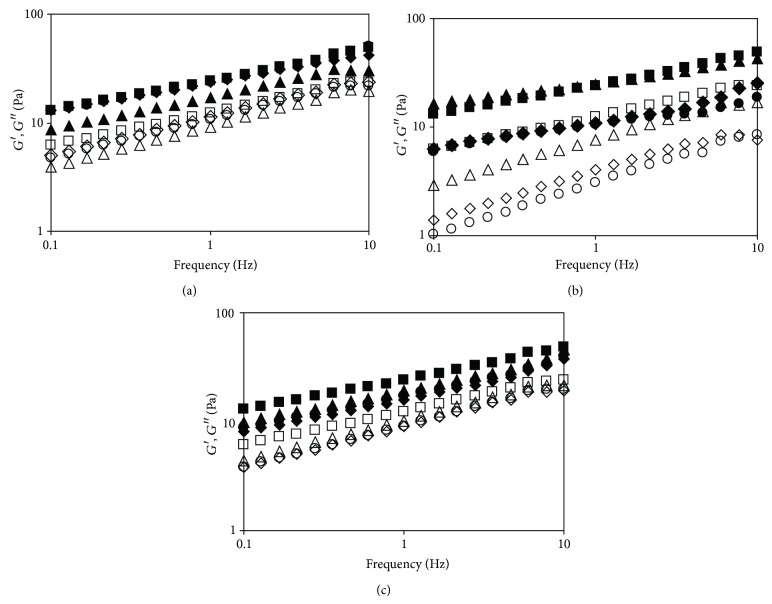
Mechanical spectra (*G*′: black markers, *G*′′: white markers) of native starch and blends with (a) low-saccharified maltodextrin, (b) medium-saccharified maltodextrin, and (c) high-saccharified maltodextrin. Maltodextrin concentration: 0—□, 1—∆, 2—*◊*, 3—○ g/100g.

The mechanical spectra shown in Figure 3 demonstrate that all the starch-maltodextrin systems behaved as weak gels. In the whole range of the frequencies studied, the values of the storage modulus (*G*′) were higher than those of the loss modulus (*G*′′). However, the storage modulus did not display a plateau that is characteristic for strong gels and depended on frequency over whole study range, with the values of tg *δ* = *G*′′/*G*′ amounting to about 0.48. The various maltodextrins added to the starch differently affected its viscoelastic properties. Low-saccharified maltodextrin in the amount of 1 g/100 g caused an apparent decrease in both moduli, as compared to the gel of native starch (Figure 3), while the other systems exhibited similar values of the storage modulus and slightly lower values of the loss modulus comparing to the gel of the native starch. Medium-saccharified maltodextrin in the amount of 1 g/100 g did not affect the storage modulus but decreased the loss modulus (Figure 3(b)). Increased amount of that maltodextrin in the system resulted in a marked decrease in the values of both moduli. For the loss modulus, the larger the decrease, the greater the amount of the maltodextrin. The values of the storage modulus for the systems with 2 and 3 g/100 g of medium-saccharified maltodextrin were similar. The gels of the systems containing high-saccharified maltodextrin showed lower values of both moduli, as compared to the gel of native starch (Figure 3(c)). The decrease was the biggest when the maltodextrin was added at the amount of 1 g/100 g. Increasing maltodextrin content in the system resulted in a much smaller decrease in both moduli, with the values of the loss modulus being similar for the blends containing high-saccharified maltodextrin at the level of 2 and 3 g/100 g, and those of the storage modulus being slightly higher for the system with 3 g/100 g of the maltodextrin. Due to the fact that starch gel forming is closely related to the association of amylose chains and retrogradation of the starch polymers [20], presence of any compounds which prevent that phenomenon results in weakening the gel structure and, consequently, decreasing *G*′ and *G*′′ moduli. In the present study, similarly to the pasting characteristic and flow behaviour, the greatest effect on the weakening gel structure and reduction of starch retrogradation had medium-saccharified maltodextrin with DP = 6. Due to a possibility of medium-saccharified starch polymers to reduce starch retrogradation, addition of them to native starches can be an alternative way to the use of stabilized starches and can be a factor that reduces bread staling.

## 4. Conclusions

Maltodextrins with varied dextrose equivalents showed different effects on the rheological properties of potato starch pastes. Medium-saccharified maltodextrin (DE = 18.4, DP = 6.0) had the greatest effect on starch pasting characteristics, flow behavior, and viscoelastic properties. The contribution of the maltodextrins to the formation of starch pastes and gels was closely associated with their degree of polymerization. High-(DP = 4.2) and medium-(DP = 6.0) saccharified maltodextrins hindered the formation of the structure of starch pastes and gels. Low-saccharified maltodextrin (DE = 10.5, DP = 10.6) added to the starch affected its rheological properties to a much smaller extent than medium-saccharified maltodextrin. This could be attributed to the fact that maltooligosaccharides of DP exceeding 6, which are able by themselves to form amylose helices, participated in the formation of the structure of starch pastes and gels. 

## Figures and Tables

**Table 1:** Pasting characteristics of native starch and blends with maltodextrins.

Sample	Pasting temperature (°C)	Peak viscosity (B.U.)	Temperature at peak viscosity (°C)	Viscosity at 96°C (B.U.)	Viscosity after 20 min at 96°C (B.U.)	Viscosity at 50°C (B.U.)	Viscosity after 10 min at 50°C (B.U.)
NS	65.8 ± 0.3	2285 ± 15	93.3 ± 0.3	2210 ± 10	1340 ± 0	1970 ± 90	1925 ± 0
NS/LSM 1	65.8 ± 0.3	1875 ± 5	96.0 ± 0.0	1870 ± 10	1320 ± 10	2070 ± 0	1980 ± 0
NS/LSM 2	65.8 ± 0.3	1635 ± 5	96.0 ± 0.0	1630 ± 0	1180 ± 0	1705 ± 15	1705 ± 15
NS/LSM 3	67.3 ± 0.3	1515 ± 5	96.0 ± 0.0	1510 ± 10	1150 ± 20	1720 ± 40	1710 ± 30
NS/MSM 1	65.5 ± 0.0	925 ± 15	73.0 ± 0.0	435 ± 15	253 ± 13	358 ± 23	360 ± 20
NS/MSM 2	65.8 ± 0.3	650 ± 10	72.8 ± 0.0	175 ± 5	90 ± 0	120 ± 0	140 ± 0
NS/MSM 3	66.0 ± 0.0	535 ± 15	72.3 ± 0.0	95 ± 5	43 ± 8	50 ± 10	55 ± 5
NS/HSM 1	65.8 ± 0.3	1955 ± 25	95.0 ± 1.0	1945 ± 15	1245 ± 15	1765 ± 65	1830 ± 30
NS/HSM 2	66.0 ± 0.0	1775 ± 15	95.0 ± 0.0	1765 ± 15	1140 ± 15	1595 ± 15	1615 ± 15
NS/HSM 3	66.0 ± 0.0	1760 ± 0	94.0 ± 0.5	1750 ± 0	1115 ± 0	1555 ± 5	1575 ± 5
LSD_0.05_	0.5	33	1.0	26	26	125	56

Mean values from three repetitions ± standard deviation.

NS: native starch, NS/LSM: native starch/low-saccharified maltodextrin (1, 2, and 3 g/100 g), NS/MSM: native starch/medium-saccharified maltodextrin (1, 2, and 3 g/100 g), NS/HSM: native starch/high-saccharified maltodextrin (1, 2, and 3 g/100 g).

LSD: least significant differences.

**Table 2:** Herschley-Bulkel model parameters of native starch paste and blends with maltodextrins.

Sample	Yield stress (Pa)	Consistency coefficient (Pa s^n^)	Flow behaviour index (−)	*R* ^2^
NS	6.07 ± 0.55	3.67 ± 0.01	0.67 ± 0.00	0.9993
NS/LSM 1	4.45 ± 0.04	2.88 ± 0.11	0.68 ± 0.01	0.9992
NS/LSM 2	4.49 ± 0.38	4.79 ± 0.02	0.58 ± 0.00	0.9993
NS/LSM 3	5.40 ± 0.27	4.63 ± 0.08	0.58 ± 0.01	0.9995
NS/MSM 1	3.01 ± 0.09	0.56 ± 0.02	0.76 ± 0.01	0.9999
NS/MSM 2	2.50 ± 0.20	0.35 ± 0.02	0.74 ± 0.02	0.9994
NS/MSM 3	1.38 ± 0.10	0.17 ± 0.03	0.76 ± 0.03	0.9982
NS/HSM 1	7.32 ± 0.01	4.61 ± 0.06	0.62 ± 0.00	0.9996
NS/HSM 2	4.89 ± 0.14	3.71 ± 0.02	0.63 ± 0.00	0.9994
NS/HSM 3	4.24 ± 0.23	2.95 ± 0.07	0.65 ± 0.01	0.9976
LSD_0.05_	0.65	0.14	0.03	

Mean values from three repetitions ± standard deviation.

NS: native starch, NS/LSM: native starch/low-saccharified maltodextrin (1, 2, and 3 g/100 g), NS/MSM: native starch/medium-saccharified maltodextrin (1, 2, and 3 g/100 g), NS/HSM: native starch/high-saccharified maltodextrin (1, 2, and 3 g/100 g).

LSD: least significant differences.
